# Extended experimental inferential structure determination method in determining the structural ensembles of disordered protein states

**DOI:** 10.1038/s42004-020-0323-0

**Published:** 2020-06-09

**Authors:** James Lincoff, Mojtaba Haghighatlari, Mickael Krzeminski, João M. C. Teixeira, Gregory-Neal W. Gomes, Claudiu C. Gradinaru, Julie D. Forman-Kay, Teresa Head-Gordon

**Affiliations:** 1 Department of Chemical and Biomolecular Engineering, University of California, Berkeley, CA 94720 USA; 2 Pitzer Center for Theoretical Chemistry, University of California, Berkeley, CA 94720 USA; 3 Department of Chemistry, University of California, Berkeley, CA 94720 USA; 4 Molecular Structure and Function Program, Hospital for Sick Children, Toronto, Ontario M5G 0A4 Canada; 5 Department of Biochemistry, University of Toronto, Toronto, Ontario M5S 1A8 Canada; 6 Department of Chemical and Physical Sciences, University of Toronto Mississauga, Mississauga, Ontario L5L 1C6 Canada; 7 Department of Bioengineering, University of California, Berkeley, CA 94720 USA; 8 Present Address: Cardiovascular Research Institute, University of California, San Francisco, CA 94158 USA

**Keywords:** Molecular modelling, Biophysical chemistry

## Abstract

Proteins with intrinsic or unfolded state disorder comprise a new frontier in structural biology, requiring the characterization of diverse and dynamic structural ensembles. Here we introduce a comprehensive Bayesian framework, the Extended Experimental Inferential Structure Determination (X-EISD) method, which calculates the maximum log-likelihood of a disordered protein ensemble. X-EISD accounts for the uncertainties of a range of experimental data and back-calculation models from structures, including NMR chemical shifts, J-couplings, Nuclear Overhauser Effects (NOEs), paramagnetic relaxation enhancements (PREs), residual dipolar couplings (RDCs), hydrodynamic radii (R_h_), single molecule fluorescence Förster resonance energy transfer (smFRET) and small angle X-ray scattering (SAXS). We apply X-EISD to the joint optimization against experimental data for the unfolded drkN SH3 domain and find that combining a local data type, such as chemical shifts or J-couplings, paired with long-ranged restraints such as NOEs, PREs or smFRET, yields structural ensembles in good agreement with all other data types if combined with representative IDP conformers.

## Abstract (web summary)

Characterising ensembles and populations of disordered structures is a challenge in structural biology. Here a Bayesian model allows solution data from NMR, fluorescence, and SAXS experiments to be synthesised in order to quantify the conformational distribution of disordered protein states.

## Introduction

Experimental techniques such as X-ray and electron crystallography and microscopy, which have traditionally excelled at determining the atomic structures of protein macromolecules and their complexes, are ill-suited for analysis of proteins with intrinsic or unfolded state disorder^1^. Instead the degree to which a simulated conformational ensemble for an intrinsically disordered protein (IDP) or unfolded state of a protein can be trusted to represent functionally relevant conformations is judged by the extent to which it conforms to the information available from solution experimental data^1,2^. Historically disordered ensemble representations were derived by utilizing the experimental data as a restraint in a molecular dynamics simulation or by choosing sets of conformations consistent with such solution data using Monte Carlo or related methods, as in the ENSEMBLE approach^3–5^.

More recently Bayesian statistical models are seen as a needed component of these approaches for disordered proteins, given the under-determined nature of solution experiments that can only measure time and/or ensemble averages and limitations of how putative ensembles are generated. Bayesian models in the protein structure context trace their origin to determine the most probable structure for folded native states using the inferential structure determination (ISD) method^6^. But to fully embrace the probabilistic interpretation of structural ensembles for disordered states, Bayesian and the related Maximum Entropy formulations account for the many different sources of uncertainty in determining the optimized structure or ensemble^7–14^. Although most of these methods have focused primarily on NMR or SAXS experimental errors and uncertainties, others have also considered the back-calculation model errors from the structure to experimental observables, or the error introduced by force field generated conformers, as summarized in a recent review^15^.

In this work, we focus on the statistical approaches for disordered states of proteins, as they raise several challenging issues in the generation and validation of structural ensembles using integrative experimental and computational techniques^16,17^. This work is distinguished from previous methodological studies^15^ as it explicitly performs single, dual, and complete joint optimization using all the experimental data for refining computational ensembles, thereby providing insights into the relative value and impact of certain data types, such as the current debate about the relationship between SAXS and smFRET^18–20^. We introduce a complete Bayesian model, the extended Experimental Inferential Structure Determination (X-EISD) method, for the statistical modeling of a wide range of experimental data types for proteins with disordered states: NMR chemical shifts and *J*-couplings^9^, homonuclear nuclear Overhauser effects (NOEs)^16,21,22^, paramagnetic relaxation enhancements (PREs)^23,24^, residual dipolar couplings (RDCs)^25,26^, hydrodynamic radii (*R*_h_)^27^, and small-angle X-ray scattering (SAXS) intensity curves^28,29^. By performing single and joint optimization using all experimental data types that probe both local and global disorder, necessary given the under-determined nature of the IDP problem^30^, we ascertain the most valuable information that takes into account uncertainties and errors provided by laboratory experiments and reported theory for back calculations.

We apply the X-EISD procedure on the unfolded state of the drkN SH3 domain because of the wide variety of experimental data types made available by the Forman-Kay and Gradinaru groups^27,31^, and which has made it popular as a test system for other ensemble scoring and refinement programs^4^. Expanding on previous work on the drkN SH3 domain, we have also introduced transfer efficiencies from single-molecule Förster resonance energy transfer (smFRET) for its unfolded state^32,33^. Starting from either an unoptimized random coil ensemble or using a reported structural ensemble of the unfolded state of the drkN SH3 domain^34^, we show through a series of single, dual and complete joint optimizations and cross-validation tests the relative influence of the different data types in scoring the putative structural ensembles. With optimization using a straightforward Markov chain Monte Carlo (MCMC) procedure on a mixed ensemble on a spectrum of disordered to ordered conformations, we show that the extensive experimental data set supports two equally probable ensembles, but each yielding an alternative structural view that can stimulate further experiments. The X-EISD Bayesian method can be downloaded and run stand-alone from a publicly available GitHub repository (https://thglab.berkeley.edu/software-and-data/) or as part of the ENSEMBLE program^5^.

## Results

### Theory

The X-EISD method is formulated as a generalized Bayesian model

1 $$\log p\left( {X,\xi |D,I} \right) = \log p\left( {X{\mathrm{|}}I} \right) + \mathop {\sum }\limits_{j = 1}^M \log \left[ {p\left( {d_j|X,\xi _j,I} \right)p\left( {\xi _j|I} \right)} \right] + C$$

where the additive constant *C* accounts for the general formulation when certain probabilities do not vary as a function of the parameters being optimized. More interestingly, $\log p\left( {X,\xi |D,I} \right)$ is the log-likelihood that the ensemble of *N* conformations $X = \left\{ {x_i} \right\}_{i = 1}^N$ are in agreement with the set of *M* experimental values $D = \left\{ {d_j} \right\}_{j = 1}^M$, given back-calculation error and experimental uncertainties {*ξ*}, and any related prior information *I*. The structural prior *p*(*X*|*I*) can be treated as either an uninformative prior or a structural prior based on Boltzmann weighting; in this work we use Jeffries uninformative prior^9^. Other Bayesian methods have primarily used a Boltzmann weighted ensemble^13^, although the general form of Bayes theorem and hence other methods acknowledge that other priors are possible. The reason we have chosen not to use Boltzmann weighted simulation conformers is that force fields are not particularly reliable for IDPs, as we have shown previously^9^. It is important to state that the prior distribution $p\left( {\xi _j|I} \right)$ represents the uncertainty for each experimental and/or back-calculation nuisance parameter *ξ*_*j*_ for data point *j*; because it reflects the variable uncertainties for each data type, the nuisance parameters are treated as a Gaussian random variable as described previously^9^. Finally, $p\left( {d_j|X,\xi _j,I} \right)$ models the experimental data point *d*_*j*_ given a set of conformers and model for *ξ*_*j*_ for each data point *j*. Applying the maximum likelihood estimator, the total probability is the sum over all data points.

A prototype EISD method was previously developed utilizing only *J*-coupling (JC) and chemical shift (CS) data for both folded proteins and IDPs^9^, whereas our current X-EISD method is now balanced across not just local, but long-range contacts (smFRET, PREs, and NOEs) and global size and shape information (SAXS and *R*_h_), to more fully utilize the experimental data types used to characterize IDPs. The JC and CS data types illustrate two general ways to formulate the probabilistic uncertainties for any experimental observable each of which utilizes different models for the back-calculation. These general forms are used to illustrate how to treat other data types.

### *J*-Couplings

The Karplus equation^35,36^ is used to back-calculate the *J* scalar coupling

2 $$J = A\left( {\left\langle {\cos \left( {\phi - \phi _o} \right)} \right\rangle } \right)^2 + B\left\langle {\cos \left( {\phi - \phi _o} \right)} \right\rangle + C$$

in which the *N* conformations provide an ensemble-averaged value of $\left\langle {\left( {\cos \left( {\phi - \phi _o} \right)} \right)^2} \right\rangle$and $\left\langle {\cos \left( {\phi - \phi _o} \right)} \right\rangle$ with respect to a reference state *ϕ*_*o*_, and Eq. (2) is used to compare with the experimentally determined value. In this case the $A(\mu _{\mathrm{A}},\sigma _{\mathrm{A}})$, $B(\mu _{\mathrm{B}},\sigma _{\mathrm{B}})$, and $C(\mu _{\mathrm{C}},\sigma _{\mathrm{C}})$ are back-calculation *ξ*_*j*_ parameters treated as Gaussian random variables for which the mean values *μ*_*j*_ and standard deviation *σ*_*j*_ are provided in the work of Vuister and Bax (*μ*_A_ = 6.51, *σ*_A_ = 0.14; *μ*_B_ = −1.76, *σ*_B_ = 0.03; *μ*_C_ = 1.60, *σ*_C_ = 0.08)^37^. The deviation of the back-calculated *J* from the given experimental *D*_*J*_ value, ${\it{\epsilon }}_{ex}^J$

3 $${\it{\epsilon }}_{ex}^J\left( {0,\sigma _{Jex}} \right) = D_J - (A\left\langle {\left( {\cos \left( {\phi - \phi _o} \right)} \right)^2} \right\rangle + B\left\langle {\cos \left( {\phi - \phi _o} \right)} \right\rangle + C)$$

is also treated as a Gaussian random variable drawn from a distribution with mean 0 and standard deviation *σ*_*Jex*_ that estimates the experimental uncertainty of the *J* measurement; in this work *σ*_*Jex*_ = 0.5 Hz based on the *J*-coupling data for the drkN SH3 domain unfolded state^27^. Hence the X-EISD method optimizes over all four sources of uncertainty

4 $$\log p\left( {J|I} \right) 	= \log p\left( {A|\mu _{\mathrm{A}},\sigma _{\mathrm{A}}} \right) + \log p\left( {B|\mu _{\mathrm{B}},\sigma _{\mathrm{B}}} \right) \\ 	\quad+ \log p\left( {C|\mu _{\mathrm{C}},\sigma _{\mathrm{C}}} \right) + \log p\left( {{\it{\epsilon }}_{ex}^J|0,\sigma _{Jex}} \right)$$

### Chemical shifts

The approach for chemical shifts, *δ*, is different, because the common back-calculators, such as SHIFTX2^38^ and SPARTA+^39^, incorporate their own internal weighting for the different components used to back-calculate *δ* for each atom type, *α*, that precludes a simple mathematical form such as the Karplus equation. For this reason, the chemical shift back-calculator is treated as a black-box model that optimizes over $q_{\delta _\alpha }$ which is treated as a Gaussian random variable with mean 0 and standard deviation $\sigma _{q_{\delta _\alpha }}$. The chemical shift function ${\it{\epsilon }}_{ex}^{\delta _\alpha }$.

5 $${\it{\epsilon }}_{ex}^{\delta _\alpha }\left( {0,\sigma _{\delta _\alpha ex}} \right) = D_{\delta _\alpha } - q_{\delta _\alpha } - \left\langle {\delta _\alpha } \right\rangle$$

is the difference between the experimental chemical shift value $D_{\delta _\alpha }$ and the average of the back-calculated shifts 〈*δ*_*α*_〉 over the ensemble, and accounting for the back-calculation error $q_{\delta _\alpha }$. In this work it is also treated as a Gaussian random variable drawn from a distribution with mean 0 and standard deviation $\sigma _{\delta _\alpha ex}$ that represents the experimental uncertainty of the chemical shift measurement; we assume a standard value of $\sigma _{\delta _\alpha ex}$ = 0.3 ppm for C, Cα, and Cβ and 0.03 ppm for H and Hα. In this work we use SHIFTX2^38^ as the back-calculation method for chemical shifts, but utilizing the published root-mean-square deviation (RMSD) we recently found for SHIFTX2 when applied to an independent protein data set^40^ of $\sigma _{q_{\delta _\alpha }}$ = 0.3–0.5 ppm for hydrogens and $\sigma _{q_{\delta _\alpha }}$ = 1.2–1.4 ppm for carbon atoms when the data is not curated and the sequence homology is low, as is true for IDPs. Hence the X-EISD method for chemical shifts optimizes over

6 $$\log p\left( {\delta _\alpha |I} \right) = \log p\left( {q_{\delta _\alpha }|0,\sigma _{q_{\delta _\alpha }}} \right) + \log p\left( {{\it{\epsilon }}_{ex}^{\delta _\alpha }|0,\sigma _{\delta _\alpha ex}} \right)$$

One could determine that the joint likelihood is ultimately a Gaussian with zero mean and standard deviation $\sigma _{q_{\delta _\alpha }} + \sigma _{\delta _\alpha ex}$. While it would be convenient to combine the two errors for a specific data type, this would hide the fact that the experimental uncertainties of a given data type can vary from measurement to measurement. Hence $\sigma _{\delta _\alpha ex}$ can be different for different measurements of many chemical shifts, even though the uncertainty of the back-calculation model to compare the experimental data to simulated structures $\left( {\sigma _{q_{\delta _\alpha }}} \right)$ does not. Separating the two errors can hopefully clarify this difference depending on the experimental data provided.

### Nuclear Overhauser effects

Characterization of NOEs for IDPs is more complex than for folded proteins due to the decreased ability to precisely assign peak values to specific nuclei due to structural ensemble averaging effects^41^. Furthermore, back-calculation of NOEs from simulation can be done to varying degrees of rigor, depending on whether or not dynamical information is available and incorporated^16^. When the conformational ensemble is derived from molecular dynamics, it is possible to fully incorporate the dynamical effects on NOEs as we have shown previously^16,21,22^. These in turn are used to calculate per-conformer estimates of the spectral density functions, allowing fairly precise back-calculation of, for example, homonuclear ^1^H–^1^H and heteronuclear ^1^H–^15^N NOEs, and R1 and R2 relaxation times^42^. When using only static structures generated with statistical coil models such as TraDES^43^ or Flexible-Meccano^44^, or any other technique where no dynamical information is available, direct back-calculation is less rigorous. In this case homonuclear NOEs can be interpreted as providing information on the distance between two spins^6,16,21^, such as the hydrogen-hydrogen distance for homonuclear ^1^H–^1^H NOEs to estimate the scaled, ensemble-averaged values of the peak intensity.

Most standard NMR spectroscopy analysis packages^45–47^ convert NOE intensities to distance restraints of varying tightness between a single pair of atoms, or pairs of atoms if the peak assignment is ambiguous. For folded proteins distance restraints are further binned into classes, such as strong restraints of <3.0 Å, medium restraints <4 Å, and weak restraints <5 Å. The observation of an NOE in a disordered state is not as closely linked to distance as in a folded state due to the dominance of dynamics and the rapid exchange between conformers. Thus, a single, generous restraint range is often given. In order to model the normal distribution in this case, the X-EISD method adopts the same approach to back-calculation as ENSEMBLE^4,5,30,34^, calculating the ensemble-averaged distance *D* from the set of *N* structures

7 $$D = \left\langle\left( {\frac{{\mathop {\sum }\nolimits_{i = 1}^N d_i^{ - 6}}}{N}} \right)^{ - 1/6}\right\rangle$$

and the deviation between experimental and back-calculation ${\it{\epsilon }}_{ex}$ is calculated as

8 $${\it{\epsilon }}_{ex}^{{\mathrm{NOE}}}\left( {0,\sigma _{{\mathrm{NOE}}ex}} \right) = D_{{\mathrm{NOE}}} - q_{{\mathrm{NOE}}} - \left\langle D\right\rangle$$

in which *q*_NOE_ and ${\it{\epsilon }}_{ex}^{{\mathrm{NOE}}}$ are Gaussian random variables, with mean 0 and standard deviations $\sigma _{q{\mathrm{NOE}}}$ and $\sigma _{{\mathrm{NOE}}ex}$, similar to that used for chemical shifts. Hence X-EISD optimizes over

9 $$\log p\left( {D_{{\mathrm{NOE}}}|I} \right) = \log p\left( {q_{{\mathrm{NOE}}}|0,\sigma _{q{\mathrm{NOE}}}} \right) + \log p\left( {{\it{\epsilon }}_{ex}^{{\mathrm{NOE}}}|0,\sigma _{{\mathrm{NOE}}ex}} \right)$$

for every distant restraint. Each experimental NOE available for the drkN SH3 domain unfolded state restrains the distance between the pair of protons to <8 or 10 Å^34^. Note that these data were derived from largely deuterated samples using long NOE mixing times, in order to increase the likelihood of NOEs representing contacts between residues far apart in sequence, and leading to longer distance restraints than typical for standard folded protein NOEs^48,49^.

Given that NOEs are formulated as distance ranges, we must consider how to model *D*_NOE_ and $\sigma _{{\mathrm{NOE}}ex}$. We use a Gaussian model to define *D*_NOE_ as the most probable distance, i.e., in the middle of the range (i.e., *D*_NOE_ = 4 or 5 Å for the drkN SH3 domain unfolded state). We then tested multiple values of $\sigma _{{\mathrm{NOE}}ex}$ to represent the distance class, i.e by dividing the experimental range of 8–10 Å by a factor of 2–5 as shown in Supplementary Fig. 1. As $\sigma _{{\mathrm{NOE}}ex}$ is further restricted, the model more closely matches one intention of the restraint—to penalize observed distances that are outside of the restraint range—however, it also results in a large range of relative probabilities within the restraint range, and might result in too strong of a bias toward an exact distance. Conversely, larger values of $\sigma _{{\mathrm{NOE}}ex}$ more closely match the expectation that all distances within the restraint range should be of roughly equal likelihood, but potentially do not sufficiently penalize distances that are outside of the restraint range (Supplementary Fig. 1). Ultimately we have found that the X-EISD optimized outcome is not particularly sensitive to the $\sigma _{{\mathrm{NOE}}ex}$ value and have defined it by dividing the experimental range of 8–10 Å by a factor of 2 ($\sigma _{{\mathrm{NOE}}ex}$ = 4 or 5 Å). Because our simple back-calculation is effectively just a comparison of ensemble-averaged simulation distances to processed experimental distance restraints, we set the back-calculation error to a small value of $\sigma _{q{\mathrm{NOE}}}$ = 0.0001 Å.

### Paramagnetic relaxation enhancements

Similar to NOEs, paramagnetic relaxation enhancements (PREs) report on ensemble- and time-averaged distances with strong dynamical contributions, but unlike NOEs the PRE signals can be measured for a much larger range of distances^25,50^. To conduct PRE experiments, a paramagnetic center must be introduced to the protein, such as through covalent bonding of a spin label, commonly MTSL for IDPs. The experiment then reports differences in the relaxation rates between the paramagnetic active sample versus its diamagnetic analog, which are converted to estimates of distances between the paramagnetic center and, most commonly, the amide protons of each residue. Multiple constructs with the tag at different locations on the protein may be used to provide several sets of restraints. As with NOEs, PREs are often converted to generic distance restraints: 25–100 Å for long distances and <10 Å for short distance restraints, and a set of medium-range distance restraints of 10–25 Å where the signal is strongest^51^. One potential issue with PREs is whether the chemical modification of the system induces different dynamics, or alters the weighting and/or introduces new structural sub-populations in the IDP ensemble^24^; at the same time, careful selection of the PRE tag and its location can be used to minimize this potential for experimental error. Hence we assume the same X-EISD model for PREs as for NOEs, with $\sigma _{q{\mathrm{PRE}}}$ = 0.0001 Å, but using $\sigma _{{\mathrm{PRE}}ex}$ that divides the experimentally-derived restraint range by 4, based on the data provided for the drkN SH3 domain unfolded state. For this data set, the medium distance PREs are centered around 12.0 Å, with most of the experimental uncertainties determined to be $\sigma _{{\mathrm{PRE}}ex}$ = 4.0 although a few PREs have $\sigma _{{\mathrm{PRE}}ex}$ ~ 11 Å.

### Residual dipolar couplings

Residual dipolar couplings (RDCs) between pairs of spins can provide useful signals for predicting local structure by inducing partial alignment of molecules in solution with magnetic field^25,26^. For IDPs, RDCs resulting from the alignment of the amide in the peptide bond are the most commonly measured and reported. Back-calculation of RDCs uses either a global alignment tensor of the static structures for the entire protein as in PALES^52^, or locally using fragments of the protein as in the local RDC calculator from the Forman-Kay group^26^. Because local back-calculation of RDCs has been shown to be able to better model experimental RDCs of disordered states when using smaller ensembles of structures^16^, we use the local RDC back-calculator from the Forman-Kay lab^26^ to get per-conformation RDCs for the amide bond vector of each residue in the target ensemble. For X-EISD scoring, we estimate the uncertainty in back-calculation error $\sigma _{q{\mathrm{RDC}}}$ = 0.9 Hz based on the standard deviation evaluated on the test set of peptides in the local RDC publication^26^. We set $\sigma _{{\mathrm{RDC}}ex}$ = 1.0 Hz given the experimental data that was deposited in the Protein Ensemble DataBank (pE-DB)^53^ for the drkN SH3 domain unfolded state^27^.

### Hydrodynamic radius

The hydrodynamic radius (*R*_h_) can be experimentally determined by calculating the translational diffusion coefficient of the macromolecule with techniques such as pulsed field gradient NMR^27^, size exclusion chromatography^54,55^, or dynamic light scattering^56^, and then using the Stokes–Einstein relationship to calculate an ensemble-averaged estimate of the *R*_h_. We use the program HYDROPRO^57^ to calculate *R*_h_, which takes static structures and uses a bead-shell model to estimate hydrodynamic properties. For X-EISD scoring, we calculate the ensemble-averaged back-calculated 〈*R*_h_〉 over the set of candidate structures, and set the experimental error to $\sigma _{{\mathrm{Rh}}ex}$ = 0.30 Å as reported in the original work on the drkN SH3 domain^27^. Because HYDROPRO is described to have +/−4% error in the estimation of *R*_h_, we assign the back-calculation error $\sigma _{q{\mathrm{Rh}}}$ = 0.8 Å given the reported experimental value of 20.3 Å^27^.

### Single-molecule fluorescence resonance energy transfer

FRET^31–33^ reports on long-range distances between two covalently bound dyes through a dipole–dipole non-radiative transfer of energy from the excited-state donor fluorophore to the ground-state acceptor fluorophore. The efficiency of energy transfer, *E*, depends sharply on the inter-fluorophore distance, $r_{D - A}$, distance:

10 $$E = \left( {1 + \left( {r_{D - A}/r_0} \right)^6} \right)^{ - 1}$$

where *r*_0_ is the Förster radius of the donor–acceptor pair. For single-molecule FRET (smFRET) measurements on IDPs and unfolded proteins, the distribution of inter-fluorophore distances is sampled much faster than the typical averaging time of the experiment (~1 ms), such that only an average FRET efficiency, 〈*E*〉, is observed^58^. The 〈*E*〉 therefore restrains the distribution of distances between two labeled residues. Multiple experiments consisting of different FRET constructs—different pairs of dyes, or dyes linked to different sites in the protein sequence—can be used to produce multiple restraints. There is a possibility that, depending on nature of the dye and the labeling site, they interact with the system and perturb its conformational landscape^19,20,59,60^, as has been seen for PREs^24^, but again can be carefully selected to minimize artifacts.

The 〈*E*〉 can be back-calculated by taking the distance measurements from static structures, calculating efficiencies, and then averaging together. Often a model is needed to account for the difference between the distance between the two residues to which dyes would be attached, and the distance between the dye centers themselves. The “scaling up” approach has been previously used to account for the FRET tags, and uses a simple polymer model to scale up the Cα–Cα distance of the native protein^61–63^:

11 $$r_{D - A} = r_{{\mathrm{C}}\upalpha - {\mathrm{C}}\upalpha }\left( {\frac{{N + N_{{\mathrm{linker}}}}}{N}} \right)^\upsilon$$

where $r_{{\mathrm{C}}\upalpha - {\mathrm{C}}\upalpha }$ is the Cα–Cα distance, *N* is the number of residues between the relevant residues, $N_{{\mathrm{linker}}}$ is the number of estimated additional amino acids, and $\upsilon$ is the Flory scaling exponent. To estimate the back-calculation uncertainty $\sigma _{q{\mathrm{FRET}}}$, we calculate the variation in back-calculated FRET efficiency that results from varying the parameters $N_{{\mathrm{linker}}}$, $\upsilon$, and *r*_0_ as discussed by Gomes and co-workers^58^ and further described in Supplementary Fig. 2. We arrive at a value of $\sigma _{q{\mathrm{FRET}}}$ = 0.007 Å, and we use a typical estimate of the experimental uncertainty of 0.02 Å for $\sigma _{{\mathrm{FRET}}ex}$.

### Small-angle X-ray scattering

Small-angle X-ray scattering (SAXS) has been a powerful tool for categorization of IDPs in their monomeric state as collapsed semi-ordered ensembles, collapsed disordered ensembles, or extended disordered ensembles^64–67^. The most well-known back-calculator from structure to SAXS intensity curves is the CRYSOL software program^28^, and for all members of the ensemble we calculate an intensity curve, *I*(*Q*), as a function of momentum transfer *Q*, and then average to obtain the SAXS observable. For X-EISD we have treated each intensity point as an independent measurement, as done in other Bayesian methods^8,13^, and scored according to the simple X-EISD formulation like individual chemical shifts via Eq. (5). The back-calculation uncertainty $\sigma _{q{\mathrm{SAXS}}} = 0.006$ is estimated by calculating overall RMSDs of the intensity points along the curve for a set of optimized ensembles. We use the experimental uncertainty estimate $\sigma _{{\mathrm{SAXS}},ex}$ = 0.0008–0.002, with the larger uncertainties defined near *Q* = 0, and decreasing toward larger values of *Q*.

But the assumption of uncorrelated or independent errors is a troublesome one for our assessment of experimental data types for X-EISD. This is because SAXS data points might be highly correlated, given close neighboring measurements in *Q*, and joint optimization might overwhelm the influence of other data types in which only one or a few observations are made, e.g., smFRET and hydrodynamic radius. Instead we have evaluated the information content in a SAXS curve based on Shannon’s sampling theorem^68–70^; for a given maximum dimension of the system *D*_max_, allows us to estimate the number of Shannon channels, *N*_s_

12 $$N_{\mathrm{s}} = D_{{\mathrm{max}}}\left( {q_{{\mathrm{max}}} - q_{{\mathrm{min}}}} \right)/\pi$$

which for the drkN SH3 domain SAXS data yields *N*_s_ ~ 3. Compared with the number of data points in the provided experimental SAXS curve of *N*_q_ = 37, this represents substantial oversampling^70^, and we have used the approach by Shevchuk and Hub^71,72^ to revise the SAXS log-likelihood score $\propto \exp ( - \left( {\frac{{\widetilde {N_s}}}{{N_q}}} \right)\frac{1}{2}\chi ^2(X))$, where χ accounts for the experimental and back-calculation errors.

### X-EISD applied to the unfolded state of the drkN SH3 domain

In order to evaluate the different local and global data types using the X-EISD Bayesian approach, we consider the unfolded state of the drkN SH3 domain^4,27,31^. The drkN SH3 domain is in slow exchange on the NMR timescale between folded and unfolded states under typical buffer conditions that are neither denaturing or stabilizing, and in this work we only consider the unfolded state. For the chemical shift, *J-*coupling, NOE, PRE, RDC, and *R*_h_ data, because of the distinct signals for the unfolded and folded states of the drkN SH3 domain, we directly use only the unfolded state NMR data. For SAXS, we use the procedure applied by Forman-Kay and co-workers previously^27^ of taking the measured experimental data for the exchanging equilibrium state, the experimental data for the stabilized folded state, and the known fraction of the folded state present at equilibrium and subtracting out the effect of the folded state to obtain experimental data for just the unfolded state of the domain. For smFRET, we ignore the peak at 〈*E*〉 = 1.0, representing the folded state, and score and optimize only using the peak at 0.55, assuming that this population represents the unfolded conformations. The total data set includes 267 chemical shifts, 47 *J*-couplings, 93 homonuclear NOE distance restraints, 68 PRE distance restraints, 28 RDCs, a SAXS intensity curve with 37 Q data points, hydrodynamic radius, *R*_h_, and smFRET efficiency data^31^.

We rank and optimize three different starting pools of structures for the unfolded state of the drkN SH3 domain. The first is a collection of ~100,000 conformations consisting of a random coil ensemble generated by gradually unfolding the folded state structure of the drkN SH3 domain^73^ with a CNS script^74,75^, including 100 folded structures and 999,900 increasingly unfolded structures (called RANDOM). These were unoptimized with respect to the experimental data. We also consider an optimized ensemble generated with the ENSEMBLE program that is comprised of 1700 conformations and is available through the pE-DB^53^ (called ENSEMBLE). This set was generated by 17 independent optimizations of 100 structures each starting from large pools of generally random structures calculated using the TraDES program^43^, including a subset that were biased to sample the non-native helical structure evident in the unfolded state based on chemical shift data. The optimization was for consistency with all of the same NMR and SAXS data types as described here, but not the smFRET efficiency data. The third starting pool (called MIXED) is described below.

**Fig. 1:**
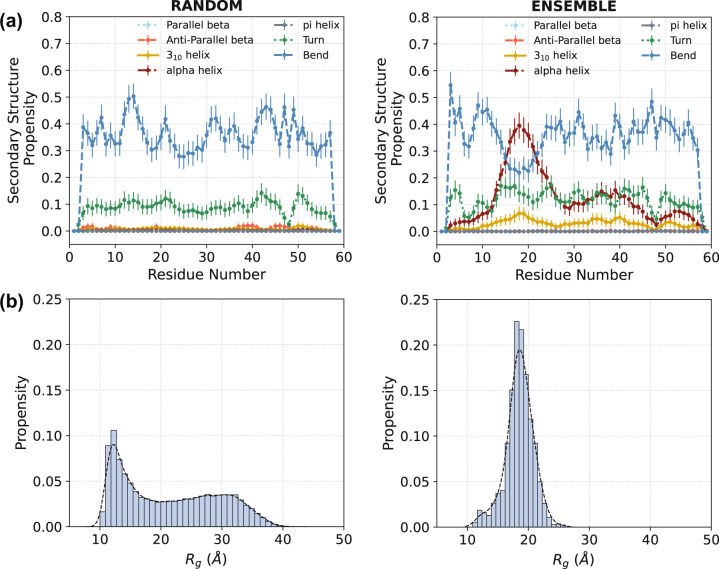
Properties of unoptimized ensembles for unfolded drkN SH3 domain. The secondary structure propensities per residue (**a**) and radius of gyration (**b**) of the drkN SH3 domain unfolded state for the unoptimized RANDOM and ENSEMBLE starting pools. Error bars are shown as ± one standard deviation for 1000 random sampling ensembles of 100 conformers each from the two starting structural pools with no X-EISD score optimization applied.

Figure 1 shows that the underlying structural picture is quite different between the RANDOM and ENSEMBLE starting pool of structures, such as the percentage of secondary structure type for each residue averaged over the pool, and global characteristics embodied in the distribution of the radius of gyration. In particular, the ENSEMBLE pool includes conformers of the drkN SH3 domain that were generated by TraDES with a bias for non-native helical propensity, and these structures were preferentially chosen by the optimization for consistency with the experimental shifts, as well as other data. Therefore, the ENSEMBLE pool is characterized by high helix propensity for residues 16–20, and some helical content over residues 30–45 and 50–55, unlike the featureless RANDOM ensemble dominated by bends and turns but no population of helical or β-sheet structure. The RANDOM starting pools exhibits a bimodal *R*_g_ distribution with 〈*R*_g_〉 of 21.2 ± 0.8 Å, representing contributions of folded, compact, and extended states sampled by the unfolding protocol, whereas the ENSEMBLE shows a very tight unimodal distribution of 〈*R*_g_〉 of 18.5 ± 0.3 Å.

Table 1 provides the X-EISD scores and RMSD error per experimental data type for the unoptimized RANDOM and ENSEMBLE starting pools of structures (see Methods), and Supplementary Table 1 shows the scores and RMSD for the complete 1700 conformer pool. Having already been refined against the full set of experimental data (except for smFRET), the ENSEMBLE starting pool appears to be a better ensemble when compared with the initial RANDOM ensemble by X-EISD score and RMSD for all data types. However, the experimental and back calculations errors (*σ*_exp_ and *σ*_q_, respectively) are larger than the RMSDs given by the ENSEMBLE pool, indicating that it is overfitted for all categories except for the smFRET data for which it was never optimized. By contrast, the smaller *σ*_exp_ and *σ*_q_ compared with the RMSDs for the RANDOM unoptimized ensemble indicate that we can refine an ensemble with higher probability than the original RANDOM structural pool, and possibly for the PREs and smFRET score for the ENSEMBLE pool as well.

**Table 1:** Evaluation of unoptimized and optimized ensembles with experimental data.

Experimental data type	UNOPTIMIZED		OPTIMIZED	
	X-EISD Score	RMSD	X-EISD Score	RMSD
**RANDOM**				
**267 CSs (ppm)**	99.6 (0.8)	0.58 (0.01)	103.6 (0.7)	0.55 (0.01)
**47 JCs (Hz)**	−82.2 (4.1)	0.91 (0.01)	−25.7 (2.4)	0.70 (0.01)
**28 RDCs (Hz)**	−59.7 (1.1)	1.23 (0.06)	−55.4 (0.6)	0.98 (0.04)
**93 NOEs (Å)**	497.3 (5.4)	4.63 (0.23)	528.5 (1.5)	3.06 (0.10)
**68 PREs (Å)**	−238.4 (186.3)	6.11 (0.74)	450.0 (4.4)	1.24 (0.12)
**smFRET <E>**	−17.4 (13.4)	0.14 (0.04)	6.9 (0.1)	0.01 (0.00)
**R**_**h**_ **(Å)**	−0.9 (0.3)	0.78 (0.30)	−0.4 (0.0)	0.14 (0.10)
**SAXS (Intensity)**	448.9 (2.9)	0.004 (0.001)	456.3 (0.4)	0.002 (0.000)
**ENSEMBLE**				
**267 CSs (ppm)**	108.7 (0.7)	0.52 (0.01)	110.1 (0.4)	0.51 (0.00)
**47 JCs (Hz)**	34.4 (1.8)	0.30 (0.02)	43.3 (0.5)	0.18 (0.01)
**28 RDCs (Hz)**	−51.8 (0.6)	0.70 (0.05)	−50.4 (0.2)	0.56 (0.03)
**93 NOEs (Å)**	517.7 (5.7)	3.80 (0.36)	539.0 (0.6)	2.33 (0.05)
**68 PREs (Å)**	0.55 (0.01)	3.33 (0.80)	458.7 (4.2)	0.92 (0.11)
**smFRET <E>**	0.70 (0.01)	0.07 (0.03)	7.0 (0.0)	0.00 (0.00)
**R**_**h**_ **(Å)**	0.98 (0.04)	0.30 (0.11)	−0.8 (0.0)	0.71 (0.04)
**SAXS (Intensity)**	3.06 (0.10)	0.004 (0.001)	457.9 (0.2)	0.001 (0.000)
**MIXED**				
**267 CSs (ppm)**	110.1 (0.9)	0.49 (0.01)	111.5 (0.5)	0.49 (0.01)
**47 JCs (Hz)**	−6.1 (7.1)	0.60 (0.04)	41.4 (0.7)	0.21 (0.01)
**28 RDCs (Hz)**	−54.7 (0.9)	0.93 (0.06)	−50.7 (0.3)	0.60 (0.03)
**93 NOEs (Å)**	514.2 (4.7)	3.91 (0.25)	539.4 (0.7)	2.28 (0.07)
**68 PREs (Å)**	155.5 (142.8)	3.89 (0.77)	458.6 (4.3)	0.92 (0.11)
**smFRET <E>**	−4.6 (8.3)	0.10 (0.04)	6.9 (0.0)	0.01 (0.00)
**R**_**h**_ **(Å)**	−0.5 (0.1)	0.25 (0.18)	−0.7 (0.0)	0.69 (0.05)
**SAXS (Intensity)**	451.9 (2.6)	0.004 (0.001)	458.0 (0.2)	0.001 (0.000)

**Fig. 2:**
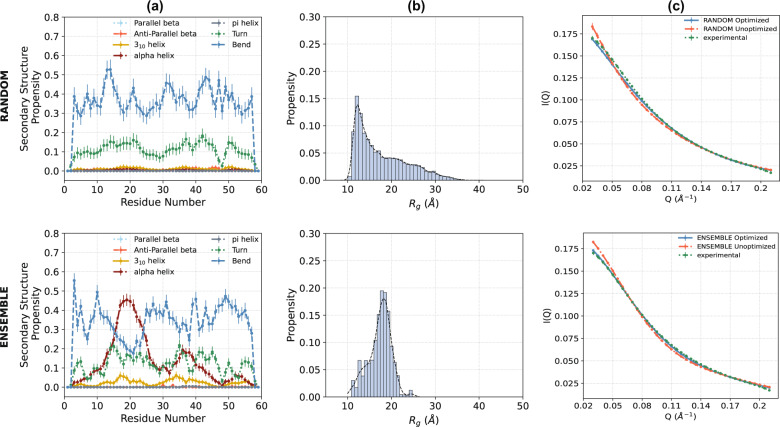
Properties of optimized ensembles for unfolded drkN SH3 domain. Results are for the optimized RANDOM and ENSEMBLE pools using all experimental data. **a** Secondary structure propensities per residue after optimization. Error bars are shown as ± one standard deviation for the secondary structure propensities among the 1000 independently drawn and optimized ensembles of 100 structures each. **b** Radius of gyration distribution after optimization. **c** SAXS intensity curves for unoptimized and optimized ensembles compared with the experimental data with corresponding errors shown with error bars.

In Table 1 and Fig. 2, we also provide the results of a MCMC maximization procedure using an X-EISD score defined as the sum of the $\log p\left( {X,\xi |D,I} \right)$ for all of the data types

13 $$acc\left( {i \to j} \right) = {\mathrm{X}}{\hbox{-}}{\mathrm{EISD}}_j \, > \, {\mathrm{X}}{\hbox{-}}{\mathrm{EISD}}_i$$

The optimized RANDOM pool is found to be positively influenced by all data types, and performs better than the original unoptimized RANDOM pool or even the original ENSEMBLE data as measured by global characteristics of the chains, i.e., NOEs and smFRET efficiency which shows greater compaction in the *R*_g_ distribution with 〈*R*_g_〉 of 17.9 ± 0.3 Å (Fig. 2). However, it is more poorly scoring in regards local structure relative to the optimized ENSEMBLE, as measured in particular by the *J*-coupling score and to a lesser extent for the chemical shifts. The optimized ENSEMBLE is better than the original ENSEMBLE with respect to all global and local data type X-EISD scores, and has a secondary assignment that favors greater amounts of helical structure for residues 16–20, 30–45, and 50–55 and an 〈*R*_g_〉 of 18.0 ± 0.1 Å.

Figure 2 also shows that the optimized RANDOM ensemble's agreement with the SAXS intensity curve is not as good as that averaged over the optimized ENSEMBLE conformers which obtains a near perfect fit to the SAXS intensity that is within experimental error. Similar conclusions are reached using a standard MCMC procedure that allows uphill moves, *acc*(*i*→*j*) = min[1, exp(*β* (X-EISD_*j*_ − X-EISD_*i*_))], using a hyperparameter *β* = 0.1 which yields ~50% acceptance rates (Supplementary Figures 3 and 4). Although the final optimized ENSEMBLE appears a better fit to the data than the optimized RANDOM ensemble, and the structural ensemble is comprised of relatively compact conformations with well-developed secondary structure in parts of the sequence, we next consider how sensitive this result is to the available conformers in the selection pool.

**Fig. 3:**
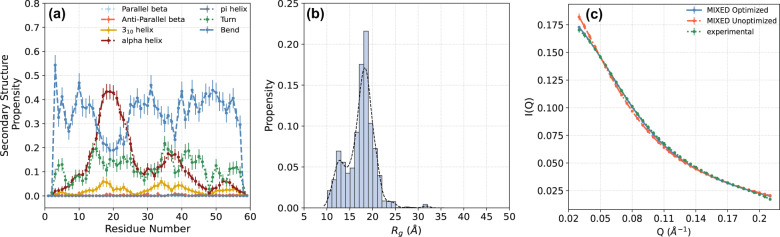
Properties of optimized MIXED pool for unfolded drkN SH3 domain. **a** Secondary structure propensities per residue, **b** radius of gyration distribution, and **c** SAXS intensity curves of the drkN SH3 domain unfolded state for the optimized MIXED ensemble. Error bars are shown as ± one standard deviation for the secondary structure propensities among the 1000 independently drawn and optimized ensembles of 100 structures each.

Therefore we created a MIXED starting pool, comprised of 50% each from the optimized RANDOM and ENSEMBLE structural pool. Table 1 shows that the X-EISD scores of this unoptimized pool are largely inferior to the two optimized parent ensembles. However, after the same MCMC optimization protocol with the X-EISD scoring function using Eq. (13), the MIXED pool shifts its composition to 24% RANDOM and 76% ENSEMBLE conformers, with better chemical shift scores that counteract the small deterioration in *J*-coupling scores that are permitted within uncertainty, relative to the optimized ENSEMBLE parent. What emerges from the optimization is a structural picture of an ensemble with largely the same local secondary structure features as the ENSEMBLE parent, but a marked decrease in the percentage of α-helix for residues 16–20, 30–45, and 50–55, and difference in global characteristics with a less compact and broader radius of gyration distribution reflective of the RANDOM pool, with an 〈*R*_g_〉 = 19.3 ± 0.5 Å and SAXS intensity profile in excellent agreement with the experiment (Fig. 3). This difference in optimized structural conformational pools between MIXED and ENSEMBLE arises from the balance among the relative changes allowed for the chemical shifts, *J*-couplings, smFRET, and NOEs, given their mix of experimental and back-calculation uncertainties. Hence, the MIXED optimized ensemble is as probable as the optimized ENSEMBLE result, but with different sub-populations of structural conformers. This provides an excellent example in which data and data processing uncertainties processed under a Bayesian formalism can yield alternative structural hypotheses that can stimulate further experiments, unlike methods that indiscriminately fit all of the experimental data.

**Fig. 4:**
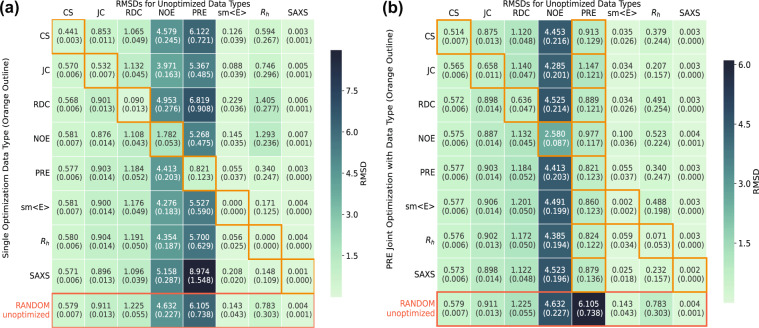
Single and dual optimization for all experimental data types. Root-mean-square deviation (RMSDs) for all data types resulting from maximizing the X-EISD score with only **a** single data type or **b** joint optimization with PREs (orange) when operating on the unoptimized RANDOM ensemble. Mean average defined over 1000 ensembles of 100 structures; numbers in parentheses are standard deviations in score among the 1000 independently optimized ensembles of each data type. The experimental and back calculations uncertainties are given in Table 1.

**Fig. 5:**
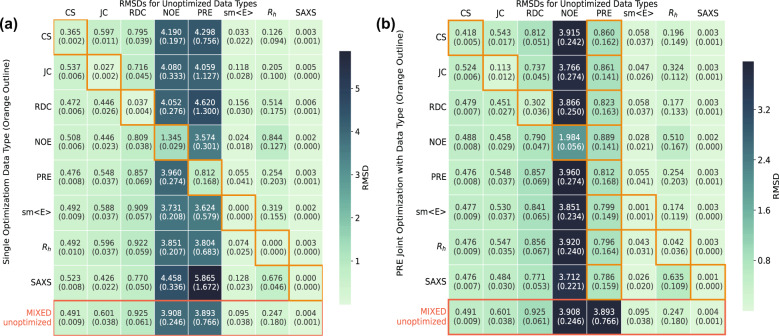
Single and dual optimization using the unoptimized MIXED ensemble. RMSDs for all data types resulting from maximizing the X-EISD score with only **a** single data type or **b** joint optimization with PREs (orange). Mean average defined over 1000 ensembles of 100 structures; numbers in parentheses are standard deviations in score among the 1000 independently optimized ensembles of each data type. The experimental and back calculations uncertainties are given in Table 1.

The X-EISD method can also provide guidance as to which experimental data type is most valuable for ensemble optimization. To show this we run the X-EISD optimization using Eq. (13) for just a single data type when operating on the unoptimized RANDOM, ENSEMBLE, and MIXED starting pools. Figures 4 and 5 show that single-mode optimization with one data type (the diagonal entries) can influence the RMSDs of unoptimized data types (off-diagonal entries) and offers interesting mutual support or discord among the experimental data types. Starting with the unoptimized RANDOM pool, the direct optimization of chemical shifts indirectly optimizes *J*-couplings, RDCs, and smFRET, while direct optimization of other local data such as *J*-couplings helps support the specific contacts that define NOEs, PREs, and smFRET (Fig. 4a). However, this is not a mutual relationship, i.e., the direct optimization of the long-ranged specific contact restraints is insufficient for indirectly benefitting chemical shifts and *J*-couplings. Hence chemical shift and *J*-coupling data are very valuable in refining a structural ensemble by providing local restrictions on how long-ranged NOEs, PREs, and smFRET contacts are formed.

There is also an asymmetric operation at play when analyzing specific long-ranged contacts such as NOEs, PREs, and smFRET, and comparing them to global shape information such as SAXS and *R*_h_. In particular, the smFRET and PRE data most significantly improve NOEs, SAXS, and *R*_h_, likely because the experimental PRE and smFRET restraints for the drkN SH3 domain unfolded state are much tighter than NOEs and more specific than SAXS and *R*_h_. A similar conclusion was reached in recent work by Gomes and co-workers that smFRET and PRE provide strong influence on IDP ensemble calculations performed on the N-terminal region of the disordered Sic1 protein^18^. While single-mode optimization with the global SAXS and *R*_h_ data offers mutual benefit to each other, they offer little indirect benefit to other localized or specific contact data types. In summary, no single optimization data type is able to bring the RMSDs to within known experimental or back-calculation uncertainties for any other data type, and joint optimization is necessary for refining the RANDOM ensemble.

The importance of mixing local information with long-ranged specific contact data can be illustrated through a dual joint optimization, which should stabilize and/or improve the RMSDs for all the remaining data types which have not contributed to the optimization. Given the single optimization results, joint optimization of *J*-couplings and PREs through a maximization procedure should improve the RMSDs, and aid the optimization across all other data types to within their expected uncertainties, a result that is supported in Fig. 4b for the RANDOM pool.

This joint optimization comes close to being statistically optimal, but ultimately the underlying RANDOM conformers are insufficient for refining the *J*-couplings to within their uncertainty. In this case the addition of other local and long-range contact data types is not useful for further refinement as the underlying RANDOM structural ensemble is not representative.

Next we consider the single and double optimization for the MIXED ensemble. In this case the unoptimized MIXED pool is a better starting point than is the RANDOM pool, and Fig. 5a shows that single optimization with PREs is nearly sufficient for generating an optimized ensemble that agrees with all experimental and back-calculation uncertainties for all data types, yielding an optimized 〈*R*_g_〉 = 19.1 ± 0.8 Å. In this case, the starting MIXED ensemble is already in sufficient agreement with the local data types, although most data types have RMSDs with large standard deviations. Figure 5b shows that joint optimization of PREs with smFRET is highly optimal for refining the MIXED ensemble for the drkN SH3 domain unfolded state to within uncertainties of all data types, again in line with that determined by Gomes and co-workers for the intrinsically disordered Sic1^18^.

**Fig. 6:**
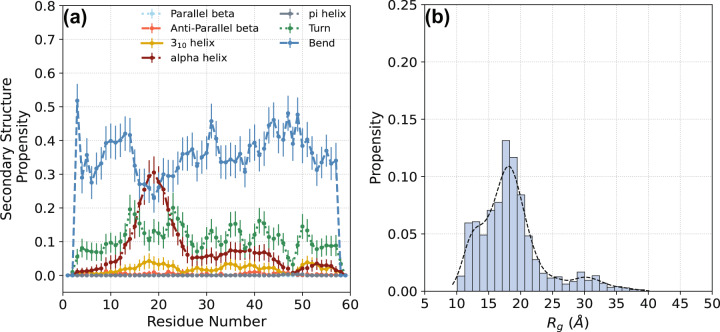
PRE and smFRET optimized MIXED pool for unfolded drkN SH3 domain. **a** Secondary structure propensities per residue and **b** radius of gyration for the dual PRE and smFRET optimized MIXED ensemble. Error bars are shown as ± one standard deviation for the secondary structure propensities among the 1000 independently drawn and optimized ensembles of 100 structures each.

In fact, the independent assessment of 〈*R*_g_〉 and secondary structure under the dual optimization scheme with PREs supports a more collapsed ensemble with greater amounts of secondary structure, and moves closer to the ENSEMBLE result (Fig. 6), with an optimized 〈*R*_g_〉 = 18.2 ± 0.4 Å. Similar conclusions are reached when optimizing on the ENSEMBLE pool of structures (Supplementary Fig. 5).

Overall, the X-EISD method allows us to state that the RANDOM structural pool is insufficient and outside the uncertainties of the local experimental data such as chemical shifts and *J*-coupling for the drkN SH3 domain unfolded state. However, while the experimental data does support local structural elements provided in the MIXED and ENSEMBLE pools, the data does not support a precise percentage of helical content, and instead ranges from 20 to 40% for the dominant helical motif at residues 16–20. More importantly, the MIXED pool supports a second population of unstructured conformers that would, as a minimum, require additional collection of more advanced NMR or smFRET experiments to probe this structural difference between the ENSEMBLE and MIXED pools.

## Discussion

We have developed a Bayesian scoring formalism for a large variety of solution experimental data types, spanning those that report on very local to very global structural information. The X-EISD approach is able to account for varying levels of uncertainty in both experiment and back-calculation for each data type, and with the very good O(N) scaling with ensemble size facilitates the high number of replicates we can perform, demonstrating the cost-effectiveness of the algorithm. One of the primary results we have demonstrated is that certain experimental data types provide more value than others for influencing the most probable disordered state ensemble, which can only be understood through a Bayesian formalism that recognizes their differences and underlying uncertainties.

Furthermore, we show how single and pairwise maximization can assess the adequacy of the underlying structural pool. For the RANDOM optimization we traced the IDP refinement to not the need for more experimental data, but better representative conformers instead. We find that dual optimization with local data such as chemical shifts and *J*-couplings combined with long-ranged restraint data such as PREs and smFRET can yield ensembles that already agree more than adequately with RDCs, NOEs, and *R*_h_ data, downplaying the need to include these data for optimization, given their larger experimental and model uncertainties. In the future, the X-EISD scoring can be utilized within more sophisticated optimization approaches, as well as operating on better designed structural ensembles, such as Boltzmann weighted ensembles derived from state-of-the-art force fields and sampling methods^16,24,76–78^.

We have shown that several equally probable disordered state ensembles are both consistent with experimental and back-calculation uncertainties for the drkN SH3 domain unfolded state domain, but differ significantly in the nature of their underlying pool of structures. While there are variable percentages of helical structure between alternate ensembles, a much stronger difference is found due to the presence or absence of completely extended conformational states, generating new hypotheses about function given their differences in weighting of distinct sub-populations of conformational states. This suggests an interesting hypothesis that there is a dynamical switching between structured and unstructured conformations in local regions of the drkN SH3 domain unfolded state, which can only be addressed with new experimental data types that are time resolved. For example, Head-Gordon and co-workers have previously found that using a relaxation description of NOEs as a dynamical constraint better agrees with experimental data for intrinsically disordered amyloid-β^16,42^, and is an important future direction for the X-EISD method to account for dynamical information.

## Methods

We examined the X-EISD scoring and RMSD for each data type to identify how large an ensemble is needed for convergence to stable mean and to determine standard deviations. We generated 1000 random sub-ensembles of sizes *N* = 2, 5, 10, 25, 50, and 100, and found that *N* = 100 is adequate for all data types and the mean sufficiently converged to allow us to provide good estimates of standard deviations without gross computational expense. We note that this is a conclusion based on computational convergence and does not reflect physical considerations of the best size of a disordered ensemble to represent reality or “maximum parsimony” designed to determine a minimum ensemble size^79^. We allow the same conformation to be selected for any number of times in any ensemble, to reflect the appropriate energy weighting or sampling of different conformational states. Supplementary Fig. 6 shows that the X-EISD score and RMSD and absolute deviation stabilizes once ensembles reach 25–100 structures, regardless of data type, and we have used the upper bound of this ensemble size.

To provide a better understanding of X-EISD scores, which will vary across all data types, we also calculate a general RMSD that allows a more intuitive measure between experimentally optimized ensembles

14 $${\mathrm{RMSD}} = \left\langle\sqrt {\frac{{\mathop {\sum }\nolimits_{i = 1}^M \left( {D_i^{{\mathrm{calc}}} - D_i^{{\mathrm{exp}}}} \right)^2}}{M}}\,\right\rangle$$

where for any data type, we take the set of M experimental values $D_i^{{\mathrm{exp}}}$ and compare them to the ensemble-averaged back-calculated values $D_i^{{\mathrm{calc}}}$. The exterior brackets reflect averaging over the repeated 1000 random sub-ensembles. We note that there is only one restraint each for 〈*E*〉 and *R*_h_, so we will generally refer to an absolute deviation from the restraint for these two data types rather than an RMSD.

We use X-EISD as a probabilistic score in a Markov Chain Monte Carlo (MCMC) optimization. We use a simple direct maximization, performing 10,000 exchange attempts to replace one conformation with another from the total pool of *N* = 100 starting structures, accepting an exchange if the new ensemble has a higher probabilistic X-EISD score than the previous. For every set of optimization conditions presented, this procedure is repeated to generate 1000 independently optimized ensembles. We perform the optimization using either a single experimental data type at a time, pairs of data types, or all data types together. Finally, we calculate properties from the optimized ensemble such as the root-mean-square *R*_g_ distribution and secondary structure content using the implementation of the DSSP algorithm^80^ within the AmberTools program cpptraj^81^.

## Supplementary information

Supplementary Information

## Data Availability

Data that support the development of X-EISD have been deposited at https://github.com/THGLab/X-EISD.

## Code availability

The code and a command-line interface are available at https://github.com/THGLab/X-EISD for the reproducibility of reported results and user accessibility for future studies.

## References

[1] Bhowmick A (2016). Finding our way in the dark proteome. J. Am. Chem. Soc..

[2] Wright PE, Dyson HJ (2015). Intrinsically disordered proteins in cellular signalling and regulation. Nat. Rev. Mol. Cell. Biol..

[3] Lindorff-Larsen K (2004). Determination of an ensemble of structures representing the denatured state of the bovine acyl-coenzyme A binding protein. J. Am. Chem. Soc..

[4] Marsh JA (2007). Improved structural characterizations of the drkN SH3 domain unfolded state suggest a compact ensemble with native-like and non-native structure. J. Mol. Biol..

[5] Krzeminski M, Marsh JA, Neale C, Choy WY, Forman-Kay JD (2013). Characterization of disordered proteins with ENSEMBLE. Bioinform.

[6] Rieping W, Habeck M, Nilges M (2005). Inferential structure determination. Science.

[7] Fisher CK, Huang A, Stultz CM (2010). Modeling intrinsically disordered proteins with bayesian statistics. J. Am. Chem. Soc..

[8] Hummer G, Kofinger J (2015). Bayesian ensemble refinement by replica simulations and reweighting. J. Chem. Phys..

[9] Brookes DH, Head-Gordon T (2016). Experimental inferential structure determination of ensembles for intrinsically disordered proteins. J. Am. Chem. Soc..

[10] Ravera E, Sgheri L, Parigi G, Luchinat C (2016). A critical assessment of methods to recover information from averaged data. Phys. Chem. Chem. Phys..

[11] Bonomi M, Camilloni C, Cavalli A, Vendruscolo M (2016). Metainference: a Bayesian inference method for heterogeneous systems. Sci. Adv..

[12] Cesari A, Gil-Ley A, Bussi G (2016). Combining simulations and solution experiments as a paradigm for RNA force field refinement. J. Chem. Theo. Comp..

[13] Kofinger J (2019). Efficient ensemble refinement by reweighting. J. Chem. Theo. Comp..

[14] Bottaro, S., Bengtsen, T. & Lindorff-Larsen, K. in *Structural Bioinformatics: Methods and Protocols* (ed. Gáspári, Z.) 219–240 (Springer US, 2020).

[15] Bonomi M, Heller GT, Camilloni C, Vendruscolo M (2017). Principles of protein structural ensemble determination. Curr. Opin. Struct. Bio..

[16] Ball KA, Wemmer DE, Head-Gordon T (2014). Comparison of structure determination methods for intrinsically disordered amyloid-beta peptides. J. Phys. Chem. B.

[17] Bottaro S, Lindorff-Larsen K (2018). Biophysical experiments and biomolecular simulations: a perfect match?. Science.

[18] Gomes, G.-N. et al. Structure and function implications of conformational ensembles consistent with smFRET, SAXS, and NMR data: the disordered protein Sic1 before and after multisite phosphorylation. *Biophys. J.* **118**, 60a (2020).

[19] Riback JA (2019). Commonly used FRET fluorophores promote collapse of an otherwise disordered protein. Proc. Natl Acad. Sci. USA.

[20] Borgia A (2016). Consistent view of polypeptide chain expansion in chemical denaturants from multiple experimental methods. J. Am. Chem. Soc..

[21] Ball KA (2011). Homogeneous and heterogeneous tertiary structure ensembles of amyloid-beta peptides. Biochem..

[22] Peter C, Daura X, van Gunsteren WF (2001). Calculation of NMR-relaxation parameters for flexible molecules from molecular dynamics simulations. J. Biomol. NMR.

[23] Milles S, Salvi N, Blackledge M, Jensen MR (2018). Characterization of intrinsically disordered proteins and their dynamic complexes: from in vitro to cell-like environments. Prog. Nucl. Magn. Res. Spect..

[24] Sasmal S, Lincoff J, Head-Gordon T (2017). Effect of a paramagnetic spin label on the intrinsically disordered peptide ensemble of amyloid-beta. Biophys. J..

[25] Newby FN (2015). Structure-free validation of residual dipolar coupling and paramagnetic relaxation enhancement measurements of disordered proteins. Biochem..

[26] Marsh JA, Baker JM, Tollinger M, Forman-Kay JD (2008). Calculation of residual dipolar couplings from disordered state ensembles using local alignment. J. Am. Chem. Soc..

[27] Choy WY (2002). Distribution of molecular size within an unfolded state ensemble using small-angle X-ray scattering and pulse field gradient NMR techniques. J. Mol. Biol..

[28] Svergun D, Barberato C, Koch MHJ (1995). CRYSOL—a program to evaluate X-ray solution scattering of biological macromolecules from atomic coordinates. J. Appl. Cryst..

[29] Sedlak SM, Bruetzel LK, Lipfert J (2017). Quantitative evaluation of statistical errors in small-angle X-ray scattering measurements. J. Appl. Cryst..

[30] Marsh JA, Forman-Kay JD (2012). Ensemble modeling of protein disordered states: experimental restraint contributions and validation. Proteins.

[31] Mazouchi A (2016). Conformations of a metastable SH3 domain characterized by smFRET and an excluded-volume polymer model. Biophys. J..

[32] Meng F (2018). Highly disordered amyloid-beta monomer probed by single-molecule FRET and MD simulation. Biophys. J..

[33] Song J, Gomes GN, Shi T, Gradinaru CC, Chan HS (2017). Conformational heterogeneity and FRET data interpretation for dimensions of unfolded proteins. Biophys. J..

[34] Marsh JA, Forman-Kay JD (2009). Structure and disorder in an unfolded state under nondenaturing conditions from ensemble models consistent with a large number of experimental restraints. J. Mol. Biol..

[35] Karplus M (1959). Contact electron‐spin coupling of nuclear magnetic moments. J. Chem. Phys..

[36] Karplus M (1963). Vicinal proton coupling in nuclear magnetic resonance. J. Am. Chem. Soc..

[37] Vuister GW, Delaglio F, Bax A (1993). The use of 1JC alpha H alpha coupling constants as a probe for protein backbone conformation. J. Biomol. NMR.

[38] Han B, Liu Y, Ginzinger SW, Wishart DS (2011). SHIFTX2: significantly improved protein chemical shift prediction. J. Biomol. NMR.

[39] Shen Y, Bax A (2010). SPARTA+: a modest improvement in empirical NMR chemical shift prediction by means of an artificial neural network. J. Biomol. NMR.

[40] Li, J., Bennett, K. C., Liu, Y., Martin, M. V. & Head-Gordon, T. Accurate prediction of chemical shifts for aqueous protein structure on “Real World” data. *Chem. Sci.* **11**, 3180–3191 (2020). 10.1039/c9sc06561j PMC8152569 34122823

[41] Novacek J, Zidek L, Sklenar V (2014). Toward optimal-resolution NMR of intrinsically disordered proteins. J. Magn. Res..

[42] Fawzi NL (2008). Structure and dynamics of the Abeta(21-30) peptide from the interplay of NMR experiments and molecular simulations. J. Am. Chem. Soc..

[43] Feldman HJ, Hogue CWV (2002). Probabilistic sampling of protein conformations: new hope for brute force?. Prot. Struct. Func. Bioinform..

[44] Ozenne V (2012). Flexible-meccano: a tool for the generation of explicit ensemble descriptions of intrinsically disordered proteins and their associated experimental observables. Bioinform.

[45] Delaglio F (1995). NMRPipe: a multidimensional spectral processing system based on UNIX pipes. J. Biomol. NMR.

[46] Guntert P (2004). Automated NMR structure calculation with CYANA. Methods Mol. Biol..

[47] Schwieters CD, Kuszewski JJ, Tjandra N, Clore GM (2003). The Xplor-NIH NMR molecular structure determination package. J. Magn. Res..

[48] Crowhurst KA, Forman-Kay JD (2003). Aromatic and methyl NOEs highlight hydrophobic clustering in the unfolded state of an SH3 domain. Biochem.

[49] Mok Y-K, Kay CM, Kay LE, Forman-Kay J (1999). NOE data demonstrating a compact unfolded state for an SH3 domain under non-denaturing conditions. J. Mol. Biol..

[50] Salmon L (2010). NMR characterization of long-range order in intrinsically disordered proteins. J. Am. Chem. Soc..

[51] Ulrich EL (2008). BioMagResBank. Nucl. Acids Res..

[52] Zweckstetter M, Bax A (2001). Single-step determination of protein substructures using dipolar couplings: aid to structural genomics. J. Am. Chem. Soc..

[53] Varadi M (2013). pE-DB: a database of structural ensembles of intrinsically disordered and of unfolded proteins. Nucl. Acids Res..

[54] Uversky VN (1993). Use of fast protein size-exclusion liquid chromatography to study the unfolding of proteins which denature through the molten globule. Biochem..

[55] Wang Y, Teraoka I, Hansen FY, Peters GH, Hassager O (2010). A theoretical study of the separation principle in size exclusion chromatography. Macromol.

[56] Nettels D (2009). Single-molecule spectroscopy of the temperature-induced collapse of unfolded proteins. Proc. Natl Acad. Sci. USA.

[57] Ortega A, Amoros D, Garcia de la Torre J (2011). Prediction of hydrodynamic and other solution properties of rigid proteins from atomic- and residue-level models. Biophys. J..

[58] Gomes G-N, Gradinaru CC (2017). Insights into the conformations and dynamics of intrinsically disordered proteins using single-molecule fluorescence. Biochim. Biophys. Acta (BBA) - Prot. Proteom..

[59] Zhang Z, Yomo D, Gradinaru C (2017). Choosing the right fluorophore for single-molecule fluorescence studies in a lipid environment. Biochim. Biophys. Acta- Biomemb..

[60] Zerze GH, Best RB, Mittal J (2014). Modest influence of FRET chromophores on the properties of unfolded proteins. Biophys. J..

[61] Meng F (2018). Highly disordered amyloid-b monomer probed by single-molecule FRET and MD simulation. Biophys. J..

[62] McCarney ER (2005). Site-specific dimensions across a highly denatured protein; a single molecule study. J. Mol. Biol..

[63] Zheng W, Borgia A, Borgia MB, Schuler B, Best RB (2015). Consistent view of polypeptide chain expansion in chemical denaturants from multiple experimental methods. J. Chem. Theory Comput..

[64] Dunker AK, Silman I, Uversky VN, Sussman JL (2008). Function and structure of inherently disordered proteins. Curr. Opin. Struct. Bio..

[65] Tompa P (2002). Intrinsically unstructured proteins. Trends Biochem. Sci..

[66] Dunker AK (2001). Intrinsically disordered protein. J. Mol. Graph. Model.

[67] Uversky VN, Dunker AK (2010). Understanding protein non-folding. Biochim. Biophys. Acta.

[68] Konarev PV, Svergun DI (2015). A posteriori determination of the useful data range for small-angle scattering experiments on dilute monodisperse systems. IUCrJ.

[69] Vestergaard B, Hansen S (2006). Application of Bayesian analysis to indirect Fourier transformation in small-angle scattering. J. Appl. Cryst..

[70] Koch M, Vachette P, Svergun D (2003). Small-angle scattering: a view on the properties, structures and structural changes of biological macromolecules in solution. Quart. Rev. Biophys..

[71] Shevchuk, R. & Hub, J. S. Bayesian refinement of protein structures and ensembles against SAXS data using molecular dynamics. *PLOS Comp. Bio.* **13**, e1005800 (2017). 10.1371/journal.pcbi.1005800 PMC5662244 29045407

[72] Bowerman S, Curtis JE, Clayton J, Brookes EH, Wereszczynski J (2019). BEES: Bayesian ensemble estimation from SAS. Biophys. J..

[73] Bezsonova I, Singer A, Choy W-Y, Tollinger M, Forman-Kay JD (2005). Structural comparison of the unstable drkN SH3 domain and a stable mutant. Biochem.

[74] Brunger AT (1998). Crystallography & NMR system: a new software suite for macromolecular structure determination. Acta Cryst. Sect. D..

[75] Brunger AT (2007). Version 1.2 of the crystallography and NMR system. Nat. Proto..

[76] Lincoff J, Sasmal S, Head-Gordon T (2019). The combined force field-sampling problem in simulations of disordered amyloid-beta peptides. J. Chem. Phys..

[77] Huang J (2017). CHARMM36m: an improved force field for folded and intrinsically disordered proteins. Nat. Meth..

[78] Rauscher S (2015). Structural ensembles of intrinsically disordered proteins depend strongly on force field: a comparison to experiment. J. Chem. Theo. Comp..

[79] Berlin K (2013). Recovering a representative conformational ensemble from underdetermined macromolecular structural data. J. Am. Chem. Soc..

[80] Kabsch W, Sander C (1983). Dictionary of protein secondary structure: pattern recognition of hydrogen-bonded and geometrical features. Biopolymers.

[81] Roe DR, Cheatham TE (2013). PTRAJ and CPPTRAJ: software for processing and analysis of molecular dynamics trajectory data. J. Chem. Theo. Comp..

