# Supplementary material for: Extended experimental inferential structure determination method in determining the structural ensembles of disordered protein states
Source: Commun Chem. 2020 Jun 9;3:74. doi: 10.1038/s42004-020-0323-0 (PMC7409953; doi:10.1038/s42004-020-0323-0)
Supplement: Supplementary file 1 — Supplementary Information [file 42004_2020_323_MOESM1_ESM.pdf]

Supplementary Information

Extended Experimental Inferential Structure Determination Method in Determining the Structural Ensembles of Disordered Protein States

James Lincoff, Mojtaba Haghighatlari, Mickael Krzeminski, João M.C. Teixeira, Gregory-Neal W. Gomes, Claudiu C. Gardinaru, Julie Forman-Kay, Teresa Head-Gordon

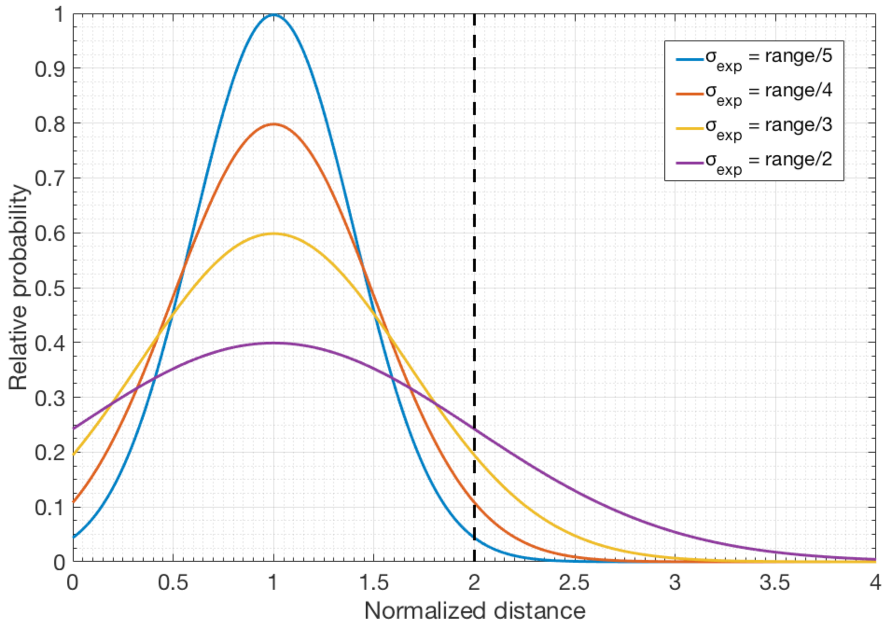

Supplementary Figure 1: *Variation in relative probabilities with different scaling of σ_{NOEex} for NOEs.* The x-axis is normalized the restraint value, 4 or 5 Å for this set of test data, such that the peak is always set to 1 in the plot, and the dashed black line represents the nominal upper bound of the restraint range.

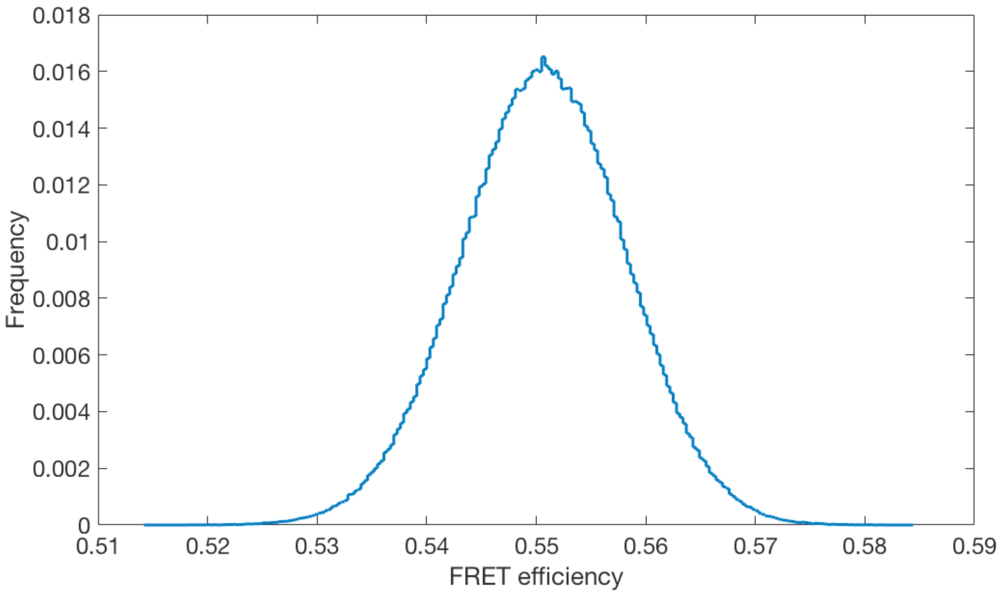

Supplementary Figure 2: *Histogram of resampled FRET efficiencies for 100-conformation best-fit ensembles for estimation of back-calculation uncertainty*. Each parameter is modeled with a normal distribution: N_{linker} with μ = 7 and σ = 3, r_0 with μ = 4.4 nm and σ = 0.2 nm, and ν with μ = 0.5 and σ = 0.05 to model slight uncertainty around the assumption of an ideal chain. The 10^6 resampled efficiencies have a standard deviation = 0.0074, which is used as the value of σ_{qFRET}.

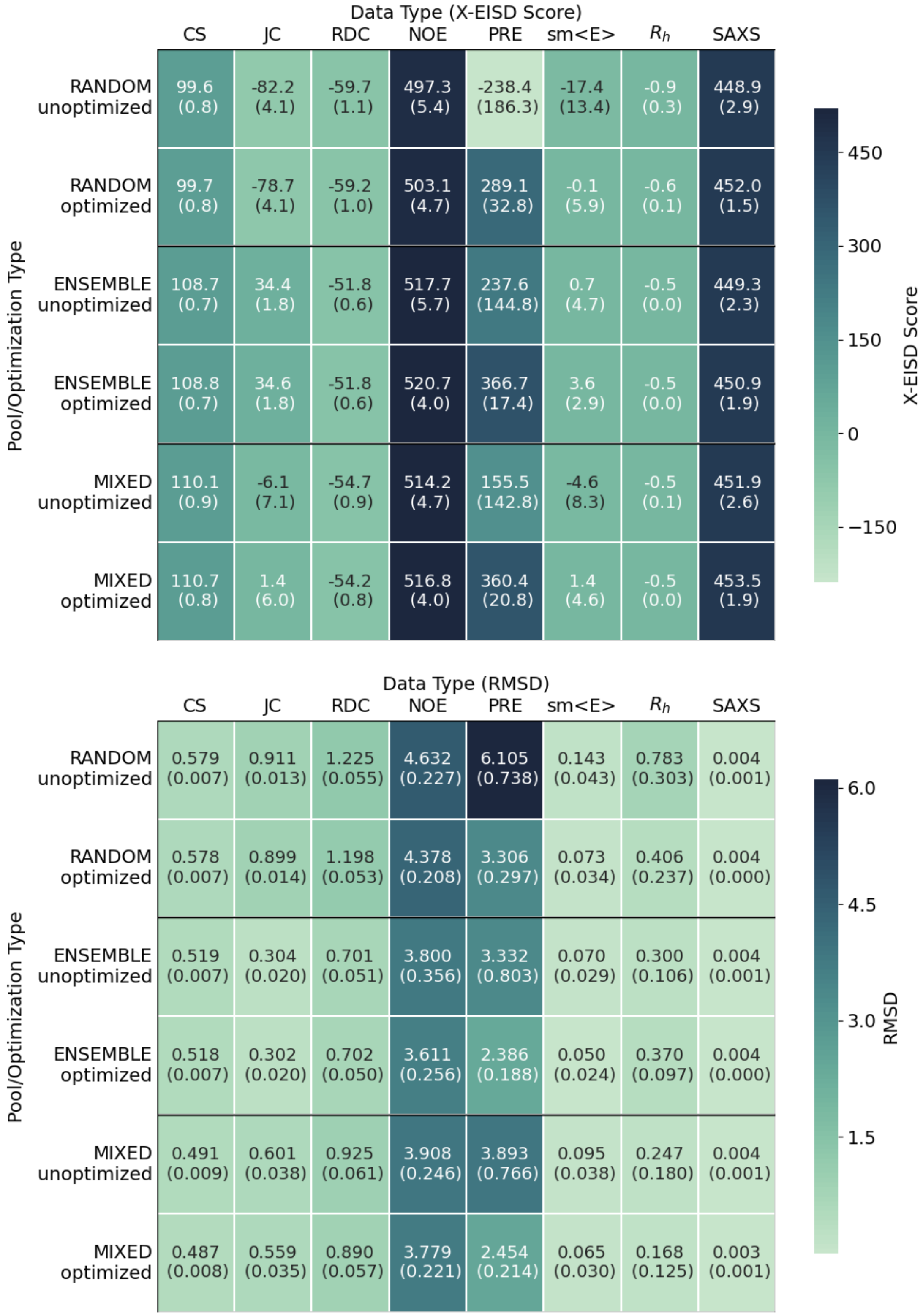

Supplementary Figure 3: *X-EISD scores and RMSDs for all experimental data types for unoptimized and optimized ensembles for the drkN SH3 domain unfolded state using* RANDOM, ENSEMBLE, and MIXED starting pools. The MCMC optimization uses $acc(i \to j) = min\left[1, exp(\beta\,(\text{X-EISD}_j - \text{X-EISD}_i))\right]$ with hyperparameter $\beta = 0.1$ which yields ~50% acceptance rates. Values in parentheses are the standard deviations that reflect variations among the 1,000 independent repeats of sub-ensembles of 100 conformations each, before and after optimization. The experimental and back calculations errors are reported in Table 1 of the main text.

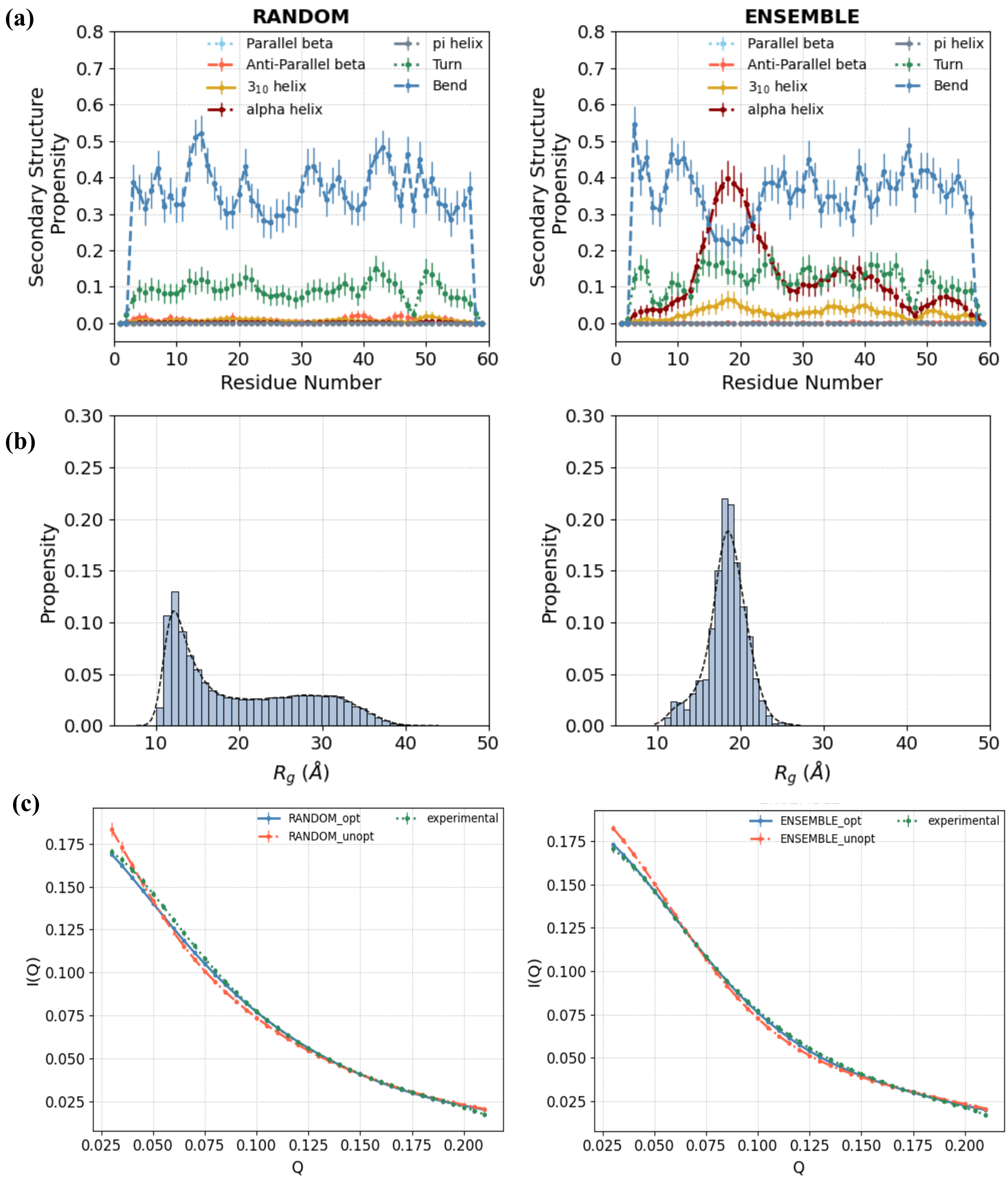

Supplementary Figure 4: *Properties of the drkN SH3 domain unfolded state for the RANDOM and ENSEMBLE ensembles compared to experiment.* (a) Secondary structure propensities per residue after optimization, (b) radius of gyration distribution after optimization, and (c) SAXS intensity curves for unoptimized and optimized ensembles compared to the experimental data with corresponding errors shown as error bars. The MCMC optimization uses $acc(i \rightarrow j) = min\left[1, exp(\beta\,(\text{X-EISD}_j - \text{X-EISD}_i))\right]$ with hyperparameter $\beta = 0.1$ which yields ~50% acceptance rates. Error bars are shown as ± one standard deviation for the secondary structure propensities among the 1,000 independently drawn and optimized ensembles of 100 structures each.

(a)

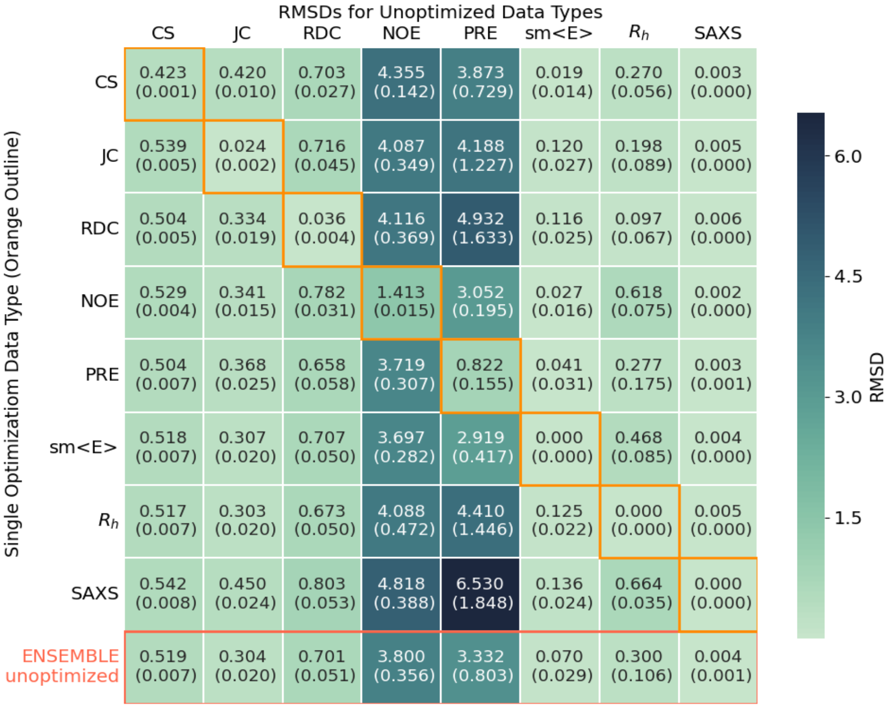

(b)

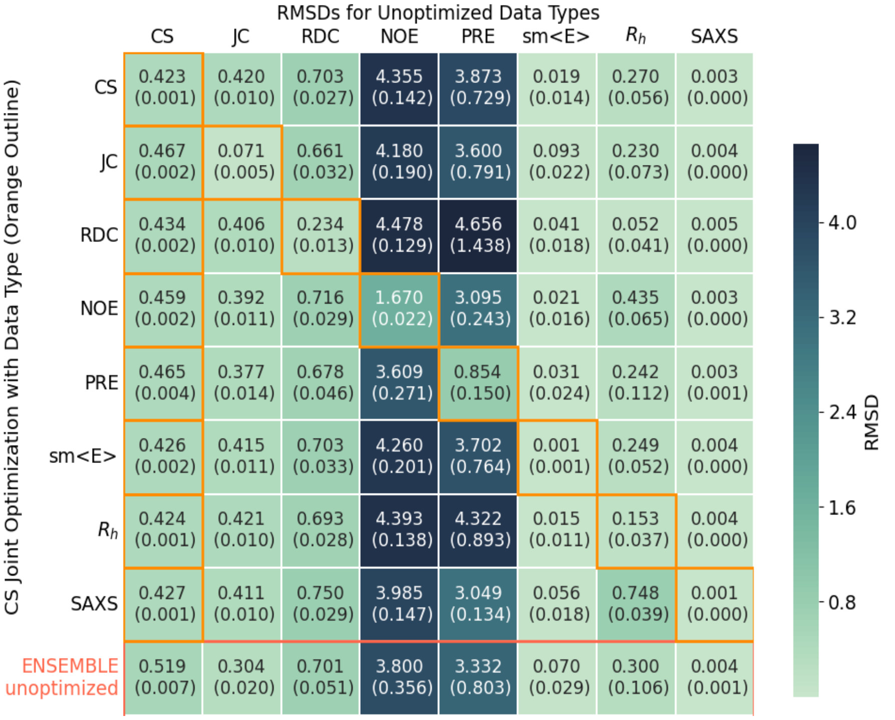

Supplementary Figure 5: *RMSDs after single and dual data type optimization operating on the unoptimized ENSEMBLE ensemble.* RMSDs for all data types resulting from maximizing the X-EISD score with only (a) single data type or (b) joint optimization with PREs (orange). Mean average defined over 1,000 ensembles of 100 structures; numbers in parentheses are standard deviations in score among the 1,000 independently optimized ensembles of each data type. The experimental and back calculations errors are reported in Table 1 of the main text.

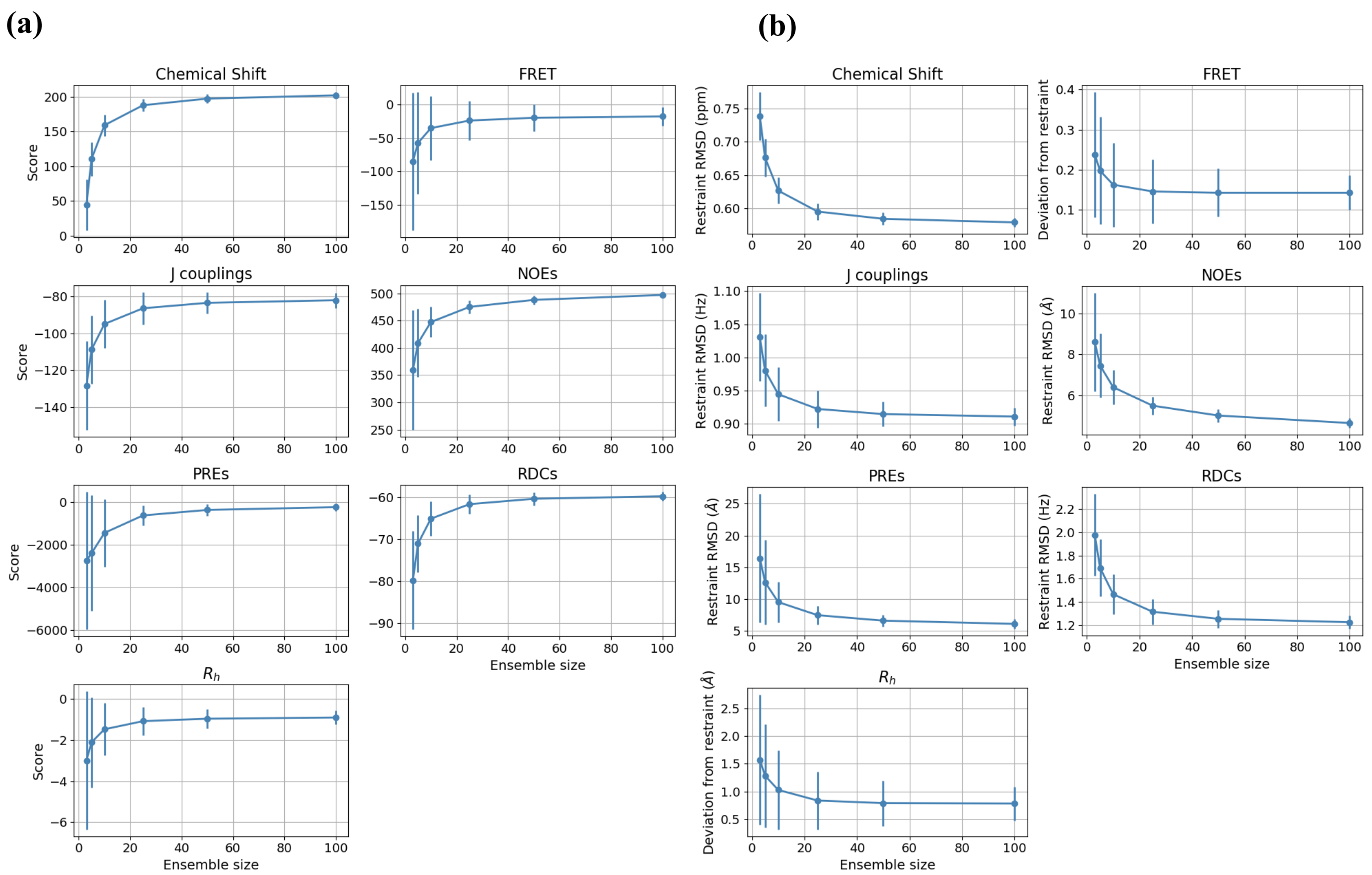

Supplementary Figure 6: Convergence of *EISD score and experimental RMSD for randomized ensembles of different sizes according to different experimental data types*. (a) Mean EISD scores and (b) restraint RMSDs are calculated across 1,000 repeated random samplings of different ensembles, with error bars representing ± one standard deviation across the 1,000 replicates. Bottom panels show the convergence of the ensemble for scores and RMSDs as a function of ensemble size. Shown are NOEs, PREs, RDCs, J couplings, chemical shifts, R_h and FRET.

Supplementary Table 1: *X-EISD scores and RMSDs for all experimental data types for unoptimized 1700 conformer ENSEMBLE pool.* The experimental and back calculations errors are reported in Table 1 of the main text.

Experimental data type	X-EISD Score	RMSD
**267 CSs (ppm)**	109.8	0.51
**47 JCs (Hz)**	37.4	0.27
**28 RDCs (Hz)**	–51.2	0.65
**93 NOEs (Å)**	535.3	2.65
**68 PREs (Å)**	375.8	2.42
**smFRET <E>**	1.4	0.07
**R_h (Å)**	–0.5	0.30
**SAXS (Intensity)**	449.5	0.004
